# Accreditation of medical education in Vietnam: From local to global excellence

**DOI:** 10.12669/pjms.38.4.5077

**Published:** 2022

**Authors:** Thuy Minh Ha, Zarrin Seema Siddiqui

**Affiliations:** 1 Thuy Minh Ha, MD, MD Education, College of Health Sciences, VinUniversity, Vietnam; 2 Zarrin Seema Siddiqui, MBBS, PhD, MD Education, College of Health Sciences, VinUniversity, Vietnam

**Keywords:** Medical education, Accreditation, Higher education, Vietnam

## Abstract

Medical education in Vietnam is going through a period of transformation. The number of medical schools is growing with increased enrollment of the students to meet the workforce needs of the country. Simultaneously, there is a need to ensure the quality of medical graduates and that there are mechanisms in place through relevant regulatory bodies.

As part of general framework, accreditation of higher education institutions is already a requirement in Vietnam with individual programs and disciplines to be accredited by the professional organizations within Vietnam or externally where appropriate. However, accreditation of medical education programs is not established as a separate entity. No medical education program in Vietnam has undergone an external evaluation but there are ongoing discussions at various forums to initiate an independent process for medical education programs. There is a consensus among stakeholders that the accreditation of medical education programs will have a potential to drive quality improvement. In this paper, we present a brief overview of the trajectory of accreditation process in Vietnam with recommendations to move forward. The journey ahead will require a coordinated approach from all stakeholders to build an accreditation system, which ensures that quality healthcare, is offered by the workforce in Vietnam.

## BACKGROUND

Medical education in Vietnam is going through a period of transformation. The number of medical schools is growing with increased enrollment of the students to meet the workforce needs of the country. There are twenty-nine medical universities in Vietnam, with an average of 400-600 medical students graduating per year at each school.^1^ This increase has also led to a demand for quality assurance and mechanisms for regulation and training of the health professionals. Ministry of Education and Training (MOET) hold primary responsibility for quality assurance of all degree and professional programs in Vietnam. Several steps are already introduced at local level and every effort is made to achieve excellence at global and international level. In this article, we present a brief overview of the accreditation process in Vietnam followed by information related to the medical education with some recommendations based on our experience and from the literature.

### Accreditation process for Higher Education Institutions in Vietnam:

At the institutional level, accreditation is a legal requirement for all higher education institutions (HEIs) with a five-year cycle. MOET has defined a set of national higher education accreditation standards adapted from the Asian University Network – Quality Assurance (AUN-QA).^2^ In all, there are twenty five standards within four domains (Table-I). Guidelines and interpretation of the criteria, including required evidence, are developed for self-evaluation and external evaluation.^3^,^4^

**Table-I:** Institutional accreditation standards (MOET, 2017).

List of domains and accreditation standards		Number of Criteria
***Domain 1. QUALITY ASSURANCE IN TERMS OF THE STRATEGY***		
1.1	Vision, mission, and culture	5
1.2	Administration	4
1.3	Leadership and management	4
1.4	Strategic management	4
1.5	Policies on education, scientific research, and community service	4
1.6	Human resource management	7
1.7	Financial and material facilities management	5
1.8	Networks and external relations	4
***Domain 2. QUALITY ASSURANCE OF THE SYSTEM***		
2.1	Internal quality assurance system	6
2.2	Internal assessment and external assessment	4
2.3	Internal quality assurance information system	4
2.4	Quality improvement	5
***Domain 3. QUALITY ASSURANCE OF FUNCTIONAL PERFORMANCE***		
3.1	Enrollment and admission	5
3.2	Design and review of curriculum	5
3.3	Teaching and learning	5
3.4	Learner assessment	4
3.5	Learner service and support activities	4
3.6	Scientific research management	4
3.7	Intellectual property management	4
3.8	Scientific research cooperation and partnership	4
3.9	Community service and connection	4
***Domain 4. PERFORMANCE RESULTS***		
4.1	Training result	4
4.2	Scientific research result	6
4.3	Community service result	4
4.4	Financial and market result	2

At the programmatic level, MOET has also defined a generic set of standards for all higher educational programs (Table-II).^5^ General Department of Education Testing and Accreditation (GDETA) was the first accreditation body established in Vietnam in 2003, which marked the formalization of a quality assurance system in the country.^6^ During the period from 2003 – 2013, GDETA solely supervised and manage accreditation activities in the education system, with including establishment of new accreditation bodies. Later, five more accreditation agencies were established in public and private sector (Table-III). These agencies undertake external evaluation and recognize education providers and programs that meet the standards set by MOET.^7^ Initially, it was planned to provide autonomy to these organization within three years’ time however, it has not happened till submission of this paper.^7^

**Table-II:** Generic set of standards for programmatic accreditation (MOET, 2016).

List of accreditation standards		Number of Criteria
1	Objectives and graduation requirements	3
2	Program summary	3
3	Curriculum structure and contents	3
4	Teaching and learning approaches	3
5	Assessment of students’ learning outcomes	5
6	Staff of lecturers and academics	7
7	Employees	5
8	Students and support given to students	5
9	Facilities and equipment	5
10	Quality improvement	6
11	Student outcomes	5

**Table-III:** List of accreditation bodies in Vietnam (June 2020).

Abbreviation	Name	Established year
VNU-CEA	Vietnam National University - Center for Education Accreditation	2013
VNU-HCM CEA	Vietnam National University - HCM Center for Education Accreditation	2013
CEA-UD	Centre for Education Accreditation - University of Danang	2015
CEA-AVU&C	Centre for Education Accreditation - Association of Vietnam Universities and Colleges	2015
VU-CEA	Vinh University - Centre for Education Accreditation	2017

For accreditation of programs, HEIs have the flexibility to follow either the national set of standards or international assessment and accreditation systems.^6^ Similarly, institutions have autonomy to select any of the national accreditation agencies, except in cases where the agency belongs to the same institution. The involvement of international agencies and boards is meant to reduce the workload for the local accreditation agencies as well as to encourage Vietnamese institutions to strive for regional and international recognition.^6^

### Medical Education in Vietnam:

Medical Doctor (MD) program is a six-year undergraduate program. The Ministries of Health (MOH) and of Ministry of Education and Training (MOET) oversee the curriculum development. MOET is responsible for promulgating the curriculum and to oversee accreditation and quality assurance like all other programs, while the content of the medical curriculum is the responsibility of and proposed by MOH.^8^

**Fig.1:**
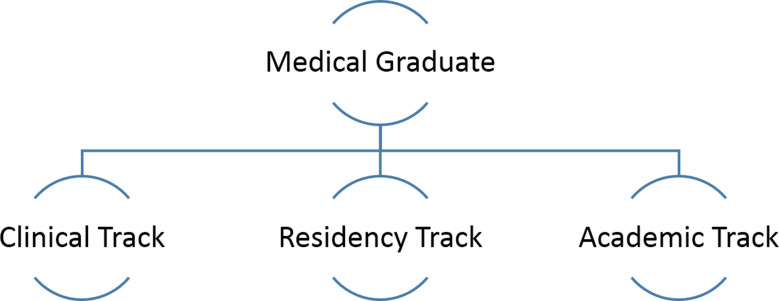
Three tracks for a medical graduate in Vietnam.

Of twenty-nine existing medical universities, there are eighteen of them in the public sectors. In collaboration with the MOET, the MOH has also defined the criteria for establishment of new schools with training program in healthcare, including competency standards for each program. A medical graduate in Vietnam can follow three tracks (Fig.1).

The clinical track trains specialists at two levels. Level-1 requires a minimum of one year of clinical experience while specialists at Level-II need a minimum of six years’ experience after completing Level-1 training.

The residency track includes research requirements alongside clinical training and the residents can assume faculty roles upon completion of their training.

The academic track leads to Master’s and PhD programs and is overseen by MOET while the other two tracks are within the domain of MOH.

MOH is striving to reform medical education for more than two decades through a consultative process and with support from donor agencies. From 1995 to 2005, the Netherlands Government supported a consortium of eight medical schools in Vietnam to introduce medical education reforms.^9^ Since 2014, World Bank has played a critical role in supporting the reforms through strengthening management competencies in the health sector and improving the competencies of primary health care.^10^ Accreditation with a vision of national and international benchmarking is currently what MOH is aiming for and is actively seeking feedback from different stakeholders.

One of the issues raised among medical education community is that the standards defined by MOET are not relevant for professional degree programs; hence, no medical program to date is accredited under the national set of standards. This implies that stakeholders need to reconsider the approach when it comes to basic medical education programs in Vietnam. We are therefore proposing some recommendations for stakeholders to consider so the process continues smoothly.

### Recommendation One: Identify the standards

World Federation of Medical Education (WFME) is an independent public organization established in 1972 by the World Health Organization and the World Medical Association.^11^ The organization has published accreditation criteria for all stages of medical education i.e., basic, postgraduate, and continuing professional development. These standards are endorsed worldwide, and relevant bodies have incorporated these standards while ensuring local values and societal needs. Other accreditation models to consider are the standards defined by the Liaison Committee on Medical Education (LCME) in the United States of America (USA), the Committee on Accreditation of Canadian Medical Schools (CACMS) in Canada, the Australian Medical Council (AMC), and the General Medical Council (GMC) in the United Kingdom.

In Asia, the WFME standards for basic medical education are adapted by many countries including Pakistan, Taiwan, South Korea, and Japan.^12^,^13^ It is therefore pertinent to learn from these countries in the region because we share the same challenges. For example, a recent study from Pakistan identified various systematic, resources and personnel related challenges.^12^ This is more important as from 2024, the Educational Commission for Foreign Medical Graduates (ECFMG) in USA will only accept applicants from a medical school accredited by an agency recognized by the WFME.^15^ This will also enable MOH, MOET and medical schools’ leadership to harmonize accreditation standards and processes for promoting excellence in medical education worldwide.

### Recommendation Two Involve all stakeholders:

A task force comprising all stakeholders be formed to assess how WFME standards can be contextualized within the Vietnamese setting. This group can include governmental, institutional, industrial representatives, physicians in academic and non-academic practice, and should also include students, residents in training and recent graduates. Participation in WFME conferences as well as local, national, and regional workshops will create awareness about quality assurance in medical education. This will also allow the participants to discuss and clarify expectations related to each standard. A similar study in Pakistan observed that there were ten WFME standards which had lower acceptability.^14^ Once standards are established, pilot trials will provide feedback to revise the process and procedures.

### Recommendation Three: Delegate the task to a national agency

Delegate procedures to an independent national agency responsible solely for medical education. Once established, the agency could seek recognition from WFME like other accreditation bodies in the region such as the Taiwan Medical Accreditation Council, Thailand Institute for Medical Education Accreditation, South Korean Institute of Medical Education and Evaluation, Indonesian Accreditation Agency for Higher Education in Health, and Japan Accreditation Council for Medical Education, and Working Committee for the Accreditation of Medical Education, Ministry of Education China.^16^ Establishing separate accreditation is foreseeable to require certain resources, but beneficial to reduce the workload for existing agencies in response to the growing demand. The Vietnam National Medical Council is already established with responsibilities for quality control of medical training and practice.^17^ Detailed scope of work for this Council is under determination, however, this can lead the accreditation process and support institutions, to get their programs recognized.

### Recommendation Four: Invest in capacity development

There is a shortage of professionals with expertise and experience in medical education at the policy-making level. Any accrediting agency will rely on experts who are able to engage with academic institutions and comply with requirements efficiently. Capacity development is therefore important so faculty can effectively use the accreditation process for quality improvement in classroom and clinical settings.

### Recommendation Five: Establish collaborative networks

Part of faculty development may be creating a community of experts who work together for mutual support, learning, and innovation on issues of accreditation. Medical education units within universities will form the foundation for this network and can be organized in a manner similar to the Partnership for Health Advancement in Vietnam.^18^

## CONCLUSION

Accreditation of medical education programs has potential to drive quality improvement in Vietnam, as the country still lags behind other countries in the region. This paper provides a brief overview of the trajectory of the accreditation process with some preliminary recommendations for next steps. The journey ahead will require a coordinated approach from all stakeholders to build an accreditation system to ensure that quality healthcare is offered in Vietnam.
